# Editorial: New trends in managing obesity in primary care

**DOI:** 10.3389/fmed.2025.1761474

**Published:** 2026-01-09

**Authors:** Camila Medeiros da Silva Mazzeti, Cain Craig Truman Clark, Bruna Paola Murino Rafacho

**Affiliations:** 1 Observatory of Chronic Conditions and Food (OCCA), Faculty of Pharmaceutical Sciences, Food and Nutrition (FACFAN), Federal University of Mato Grosso do Sul (UFMS), Campo Grande, Brazil; 2 College of Life Sciences, Birmingham City University, Birmingham, United Kingdom

**Keywords:** chronic care model (CCM), disease management, family medicine and primary care, overweight and obesity, primary health care

Obesity is one of the most complex and challenging chronic conditions of the 21st century. Multifactorial, persistent, and profoundly influenced by social, economic, environmental, and cultural determinants, it permeates all dimensions of public health. However, although widely known as a global epidemic, obesity remains underdiagnosed, underfunded, and, above all, invisible as a disease within health systems (1, 2). The result is a cycle of late, fragmented, and unequal interventions, failing to address the root of the problem, which often makes care, wholly or partly, ineffective (3, 4).

Primary Health Care (PHC) is undoubtedly the most appropriate sector to break this cycle. Due to its attributes of accessibility, longitudinality, comprehensiveness, and coordination of care, PHC is the natural space to address obesity in its multiple and complex dimensions, from health promotion to prevention, from early diagnosis to continuous monitoring of those already living with the condition (5, 6). More than just an entry point, PHC should be the structuring axis of obesity care, articulating clinical, community, and intersectoral actions that facilitate a humanized and effective therapeutic path (7).

The studies presented in this Research Topic reinforce this urgency. Tang et al. demonstrate that, among primary healthcare physicians in China, there remain significant gaps in knowledge, attitude, and practice regarding obesity. Although 75% recognize its health impacts, less than half have adequate mastery of diagnosis and management. The finding is emblematic: even at the most basic levels of the system, there is a lack of preparedness to deal with a condition that should be central to daily practice.

Zhang et al. complement this perspective by describing that simple indicators, such as waist circumference, might be better predictors of glycemic dysregulation in elderly diabetics than body mass index (BMI). The study reinforces that it is opportune to review health diagnostic parameters based solely on BMI, a useful indicator in primary health care practice, but limited in addressing all the dimensions that permeate a person with obesity or chronic diseases. The diagnosis should consider physical, biochemical, behavioral, and psychological contexts, reflecting the complexity of the phenomenon. Building an international consensus on the definition and assessment of obesity is urgently needed, so that not only body weight is recognized, but also the suffering, metabolic risk, and functional impact that accompany this condition.

The human dimension of care is highlighted by Fang et al., who explore the experience of patients after bariatric surgery in China. The study reveals gaps in preparation for hospital discharge, self-management, and social support, highlighting the essential role of a multidisciplinary approach and digital support systems in ensuring postoperative follow-up. Surgery alone does not end the treatment of obesity: it must be understood as a step within a continuous care pathway, articulated with primary care and community support.

In the United States, Holtrop et al. present the perspective of teams and patients on new anti-obesity medications, especially GLP-1 receptor agonists. The interviews show enthusiasm for clinical results, but also concerns about access, cost, prescribing criteria, and the ethical and organizational implications of these pharmacological treatments. Therapeutic progress is undeniable, but it cannot occur in isolation from the structure of public health systems, which risks widening inequalities and medicalizing suffering.

Alsuhibani et al. expand on this reflection by analyzing data from Medicaid, the American health program for low-income families and individuals, between 1999 and 2023, showing a 6.7% increase in prescriptions for anti-obesity medications and a seemingly inexorable rise in public spending, amounting to ~77 million percent. This escalation underscores the economic impact of relying exclusively on pharmacotherapy and highlights the need for public policies that strike a balance between innovation, sustainability, and equity. The results bring important reflection on the excessive medicalization of obesity, the elitism of treatment, and the perpetuation of “fatphobia” (stigma and discrimination of obesity).

Alhowiti et al., in a study conducted in Saudi Arabia, found that more than half of GLP-1 users discontinued treatment due to cost or unavailability. That use was predominantly focused on aesthetic weight loss rather than clinical improvement of comorbidities. The study reveals an underlying problem: unequal access and decontextualized use of pharmacological therapies, often disconnected from clinical protocols, nutritional re-education programs, or multidisciplinary support. Despite the optimism surrounding the innovation of these medications, an international consensus on a prescription protocol, restriction on use without prescription, price regulation, and feasibility of application in different contexts and age groups is imperative.

Finally, two studies examine the use of complementary and alternative practices in healthcare. He et al. and Wang et al. present evidence and discuss the effects of acupuncture in menopausal women and Traditional Chinese Medicine in children, respectively, highlighting benefits and indicating the remaining gaps for the use of non-conventional therapies for the management of obesity.

The present edition reveals a contrasting scenario of obesity care, where science and technology are rapidly advancing, producing highly effective medications, digital devices, and increasingly safe surgical procedures. However, concomitantly, health practices are limited in organizing a comprehensive, equitable, and humanized response, depending on the technology incorporated. PHC, which should be at the core of this response, is often relegated to a secondary role, lacking resources, protocols, and recognition of obesity as a priority disease.

Indeed, PHC represents a key process for prevention, early identification of risks, promotion of healthy habits, ensuring longitudinal follow-up, and avoiding relapses. PHC might transform the consultation services into a space of welcoming, listening, and rebuilding autonomy for people with obesity. Future research should aim not only to refine detection and treatment but also to understand and integrate lifestyles, relationships between people, food, and living spaces. Making primary health care visible, strengthened, and committed is the most urgent step for obesity care to move beyond palliative measures and become transformative.
